# Assessing the efficacy and safety of mycophenolate mofetil versus azathioprine in patients with autoimmune hepatitis (CAMARO trial): study protocol for a randomised controlled trial

**DOI:** 10.1186/s13063-022-06890-w

**Published:** 2022-12-13

**Authors:** Romée J. A. L. M. Snijders, Anna E. C. Stoelinga, Tom J. G. Gevers, Simon Pape, Maaike Biewenga, Robert C. Verdonk, Hendrik J. M. de Jonge, Jan Maarten Vrolijk, Sjoerd F. Bakker, Thomas Vanwolleghem, Ynto S. de Boer, Martine A. M. C. Baven Pronk, Ulrich H. W. Beuers, Adriaan J. van der Meer, Nicole M. F. van Gerven, Marijn G. M. Sijtsma, Bart J. Verwer, Ingrid A. M. Gisbertz, Maartje Bartelink, Floris F. van den Brand, Kerem Sebib Korkmaz, Aad P. van den Berg, Maureen M. J. Guichelaar, Khalida Soufidi, Amar D. Levens, Bart van Hoek, Joost P. H. Drenth, on behalf of the Dutch Autoimmune Hepatitis Working Group

**Affiliations:** 1 Department of Gastroenterology and Hepatology, Radboud University Medical Center, Nijmegen, The Netherlands; 2 European Reference Network RARE-LIVER, Hamburg, Germany; 3 Department of Gastroenterology and Hepatology, Leiden University Medical Center, Leiden, The Netherlands; 4 Division of Gastroenterology and Hepatology, Department of Internal Medicine, Maastricht University Medical Center, Maastricht, The Netherlands; 5 Nutrim School for Nutrition and Translational Research in Metabolism, Maastricht University, Maastricht, The Netherlands; 6 Department of Gastroenterology and Hepatology, St. Antonius Hospital, Nieuwegein, The Netherlands; 7 Department of Gastroenterology and Hepatology, Jeroen Bosch Hospital, ‘s Hertogenbosch, The Netherlands; 8 Department of Gastroenterology and Hepatology, Rijnstate Hospital, Arnhem, The Netherlands; 9 Department of Gastroenterology and Hepatology, Elisabeth-Tweesteden Hospital, Tilburg, The Netherlands; 10 Department of Gastroenterology and Hepatology, Antwerp University Hospital, Edegem, Belgium; 11 Department of Gastroenterology and Hepatology, Amsterdam University Medical Centers, location VU University Medical Center, Amsterdam, The Netherlands; 12 Department of Gastroenterology and Hepatology, Groene Hart Hospital, Gouda, The Netherlands; 13 Department of Gastroenterology and Hepatology, Amsterdam University Medical Centers, location Academic Medical Center, Amsterdam, The Netherlands; 14 Department of Gastroenterology and Hepatology, Erasmus University Medical Center, Rotterdam, The Netherlands; 15 Department of Gastroenterology and Hepatology, Rode Kruis Hospital, Beverwijk, The Netherlands; 16 Department of Gastroenterology and Hepatology, St. Jansdal Hospital, Harderwijk, The Netherlands; 17 Department of Gastroenterology and Hepatology, Spaarne Gasthuis, Haarlem, The Netherlands; 18 Department of Gastroenterology and Hepatology, Hospital Bernhoven, Uden, The Netherlands; 19 Department of Gastroenterology and Hepatology, Deventer Hospital, Deventer, The Netherlands; 20 Department of Gastroenterology and Hepatology, OLVG Oost, Amsterdam, The Netherlands; 21 Department of Gastroenterology and Hepatology, IJselland Hospital, Capelle aan den Ijssel, The Netherlands; 22 Department of Gastroenterology and Hepatology, University Medical Center Groningen, Groningen, The Netherlands; 23 Department of Gastroenterology and Hepatology, Medisch Spectrum Twente, Enschede, The Netherlands; 24 Department of Gastroenterology and Hepatology, Zuyderland, Heerlen, The Netherlands; 25 Department of Clinical Pharmacy and Toxicology, Leiden University Medical Center, Leiden, The Netherlands

**Keywords:** Autoimmune hepatitis, Azathioprine, Mycophenolate mofetil, First-line treatment, Induction therapy, Randomized controlled trial, Remission, Biochemical remission, Phase IV trial

## Abstract

**Background:**

Currently, the standard therapy for autoimmune hepatitis (AIH) consists of a combination of prednisolone and azathioprine. However, 15% of patients are intolerant to azathioprine which necessitates cessation of azathioprine or changes in therapy. In addition, not all patients achieve complete biochemical response (CR). Uncontrolled data indicate that mycophenolate mofetil (MMF) can induce CR in a majority of patients. Better understanding of first-line treatment and robust evidence from randomised clinical trials are needed. The aim of this study was to explore the potential benefits of MMF as compared to azathioprine, both combined with prednisolone, as induction therapy in a randomised controlled trial in patients with treatment-naive AIH.

**Methods:**

CAMARO is a randomised (1:1), open-label, parallel-group, multicentre superiority trial. All patients with AIH are screened for eligibility. Seventy adult patients with AIH from fourteen centres in the Netherlands and Belgium will be randomised to receive MMF or azathioprine. Both treatment arms will start with prednisolone as induction therapy. The primary outcome is biochemical remission, defined as serum levels of alanine aminotransferase and immunoglobulin G below the upper limit of normal. Secondary outcomes include safety and tolerability of MMF and azathioprine, time to remission, changes in Model For End-Stage Liver Disease (MELD)-score, adverse events, and aspects of quality of life. The study period will last for 24 weeks.

**Discussion:**

The CAMARO trial investigates whether treatment with MMF and prednisolone increases the proportion of patients in remission compared with azathioprine and prednisolone as the current standard treatment strategy. In addition, we reflect on the challenges of conducting a randomized trial in rare diseases.

**Trial registration:**

EudraCT 2016-001038-91. Prospectively registered on 18 April 2016.

**Graphical Abstract:**

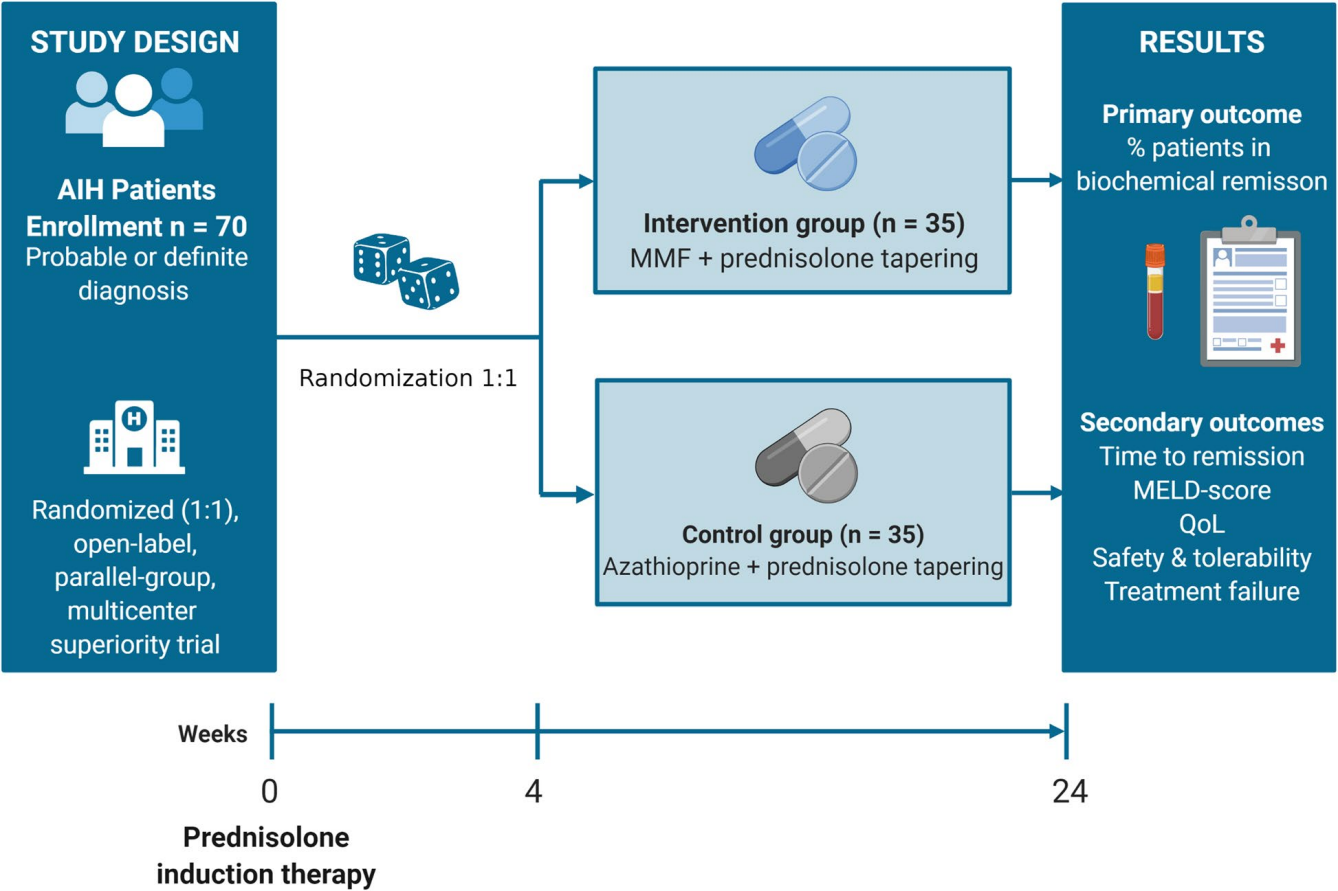

**Supplementary Information:**

The online version contains supplementary material available at 10.1186/s13063-022-06890-w.

## Background

Autoimmune hepatitis (AIH) is a severe chronic liver disease associated with cirrhosis and liver failure [1, 2]. AIH is a rare disease, its prevalence is estimated at 17–23 cases per 100,000 inhabitants in Europe [3, 4]. It is characterised by a spectrum of clinical manifestations and may lead to complications of progressive liver disease, including cirrhosis, portal hypertension, and hepatocellular carcinoma. Untreated AIH has very poor outcomes and is usually fatal [2, 5].

Immunosuppressive therapy consisting of azathioprine and corticosteroids has emerged as the mainstay of active autoimmune hepatitis management. This has greatly improved survival rates [6–8]. Normalisation of transaminases is achieved in 65–70% after 6 months, irrespective of prednisone dosage [9]. However, almost 20% of patients experience adverse effects (AEs) with azathioprine treatment, such as nausea, vomiting, arthralgia, skin rash and fever, which necessitate cessation of treatment in approximately 15% of patients in the first year [10, 11]. In recent years, mycophenolate mofetil (MMF) has been explored as an alternative option to standard care in AIH treatment. Most studies focused on the use of MMF as second-line rescue therapy in AIH patients who were either intolerant or refractory to azathioprine. MMF seems to be generally better tolerated than azathioprine. Several retrospective studies reported remission rates, which varied from 25 to 100% (Table 1) [12–25]. This has led to recommendations in the European Association of the Study of the Liver (EASL) guidelines and the European Society for Paediatric Gastroenterology Hepatology and Nutrition (ESPGHAN) position statement of MMF as second-line treatment for patients who do not respond to or do not tolerate azathioprine [26, 27]. Meanwhile, MMF has aroused interest as an alternative option for AIH induction therapy. A prospective uncontrolled study explored the efficacy of MMF in treatment-naive AIH patients [28]. Fifty-nine treatment-naive AIH patients received open-label MMF at a final dose of 1.5–2 g daily combined with a standardised prednisolone tapering scheme. Eighty-eight per cent of patients had an initial clinical and biochemical response with normalisation of serum aspartate transferase (AST), alanine transferase (ALT) and immunoglobulin gamma (IgG) levels. While the data on MMF in AIH patients are promising, randomised clinical trials are needed to generate high-quality evidence.

**Table 1:** Studies of MMF in AIH

Author	Year	Type of study	Patients, ***n***	Population (age group, variant, cirrhosis, indication for MMF)	Biochemical remission	Definition endpoint
**Richardson** [22]	2000	Retrospective	7	Population: adult and children Variant: *n*=0 Cirrhosis: *n*=1 Indication: refractory disease (*n*=4), intolerance AZA (*n*=3)	71%	Normalisation of ALT at 3 months
**Devlin** [24]	2004	Retrospective	5	Population: adult Variant: *n*=0 Cirrhosis: *n*=2 Indication: refractory disease (*n*=3), side-effects (*n*=1), other reasons (*n*=1)	100%	Normalised ALT at any timepoint
**Chatur** [21]	2005	Retrospective	16 (11/16 on MMF monotherapy)	Population: adult Variant: *n*=0 Cirrhosis: unknown^a^ Indication: unknown^a^	64%	Normalisation of serum aminotransferases at any timepoint
**Czaja** [25]	2005	Retrospective	8	Population: adult Variant: *n*=0 Cirrhosis: *n*=1 Indication: treatment naïve (*n*=1), incomplete response (*n*=4), treatment failure (*n*=2), multiple relapses (*n*=1)	0%	Normalisation of AST, bilirubin, and IgG at any timepoint
**Inductivo-Yu** [20]	2007	Retrospective	15	Population: adult Variant: *n*=2 Cirrhosis: *n*=8 Indication: biochemical or histologic non-response (*n*=11), significant side effects (*n*=14)	73%	Normalisation of ALT at unknown time point
**Hlivko** [19]	2008	Retrospective	29	Population: adult Variant: unknown^a^ Cirrhosis: unknown^a^ Indication: intolerance or nonresponse (*n*=12 ➔ 9 prednisone + MMF; 1 MMF alone; 1 prednisone + MMF + tacrolimus; 1 MMF + cyclosporine), first-line therapy (*n*=17)	55%	Resolution of symptoms, reduction in serum aminotransferase levels to <2 ULN, normalisation of serum bilirubin and γ-globulin levels (and improvement in liver histology to normal or only mild portal hepatitis) at unknown time point
**Hennes** [18]^**b**^	2008	Retrospective	36	Population: unknown Variant: unknown Cirrhosis: unknown Indication: side effects (*n*=28), insufficient response (*n*=9), pregnancy (*n*=1)	39%	AST <2x ULN at unknown timepoint
**Wolf** [17]	2009	Retrospective	16	Population: unknown Variant: *n*=4 Cirrhosis: unknown Indication: intolerance (*n*=7), refractory disease (*n*=6), other reasons (*n*=3)	31%	Reduction of ALT from greater than twice normal to less than twice normal
**Baven-Pronk** [15]	2011	Retrospective	45	Population: adult Variant: (*n*=15) Cirrhosis: (*n*=24) Indication: AZA-non-responders (*n*=22), AZA-intolerance (*n*=23)	47%	Normalisation of AST and/or ALT at any timepoint after starting MMF
**Zachou** [28]	2011	Prospective	59	Population: adult + children Variant: *n*=0 Cirrhosis at presentation: *n*=14 Indication: treatment-naïve (*n*=59)	88%	Normalisation of AST, ALT, and γ-globulins within 12 months
**Jothimani** [16]	2014	Retrospective	20	Population: adult Variant: *n*=3 Cirrhosis: *n*=5 Indication: AZA intolerance (*n*=18), refractory disease (*n*=2)	74%	Normalisation of ALT and/or AST at any timepoint
**Zachou** [29]	2016	Prospective	109	Population: adult + children Variant: *n*=0 Cirrhosis at presentation: *n*=26 Indication: treatment-naïve (*n*=109)	72%	Normalisation of ALT, AST, and IgG, symptoms improved or disappeared and liver histology, if performed, showed minimal or no inflammation at 3 months treatment
**Roberts** [14]	2018	Retrospective	105	Population: adult Variant: *n*=0 Cirrhosis: *n*=38 Indication: suboptimal response (*n*=42), treatment intolerance (*n*=63)	60%	Normalisation of ALT, AST, and IgG, with or without normal liver histology within the first 2 years of treatment
**Nicoll** [13]	2019	Retrospective	105	Population: adult Variant: *n*=0 Cirrhosis: *n*=38 Indication: intolerance (*n*=63), nonresponse (*n*=42)	60%	ALT, AST and IgG <ULN, with or without normal liver histology, within the first 2 years of treatment
**Giannakopoulos** [12]	2019	Retrospective	22	Population: adult Variant: *n*=0 Cirrhosis: *n*=6 Indication: intolerance (*n*=14), non-response (*n*=5), intolerance + non-response (*n*=3)	45%	Normalisation of ALT and AST within 3 to 30 weeks
**Liberal** [23]	2021	Retrospective	18	Population: adult Variant: *n*=0 Cirrhosis: *n*=4 Indication: intolerance (*n*=9), refractory disease (*n*=9)	39%	Normalisation of AST, ALT, and IgG at 12 months
**Dalekos** [30]	2021	Prospective	32	Population: adult + children Variant: *n*=0 Cirrhosis: *n*=6 Indication: treatment-naïve (*n*=32)	93.8%^c^	Normalisation of AST, ALT, and IgG at 6, 12 months, and at the end of follow-up

*AIH* autoimmune hepatitis, *ALT* alanine aminotransferase, *AST* aspartate aminotransferase, *AZA* azathioprine, *HAI* Hepatitis Activity Index, *IgG* immunoglobulin G, *ULN* upper limit of normal, *MMF* mycophenolate mofetil

^a^Patients with MMF: no separate baseline data ^b^full text not available ^c^at 6 months

## Methods

### Design and methods

The trial protocol is written in accordance with the Standard protocol items: recommendation for interventional trials (SPIRIT) guidelines [31] (see Table 4 and the Additional file 1: SPIRIT checklist).

### Study aim

The CAMARO trial aims to investigate whether MMF and prednisolone are superior to azathioprine and prednisolone in inducing remission in treatment-naive AIH patients. The hypothesis is that MMF will be more effective than azathioprine in inducing remission, both combined with a standard prednisolone schedule.

### Study design and recruitment

**Fig. 1:**
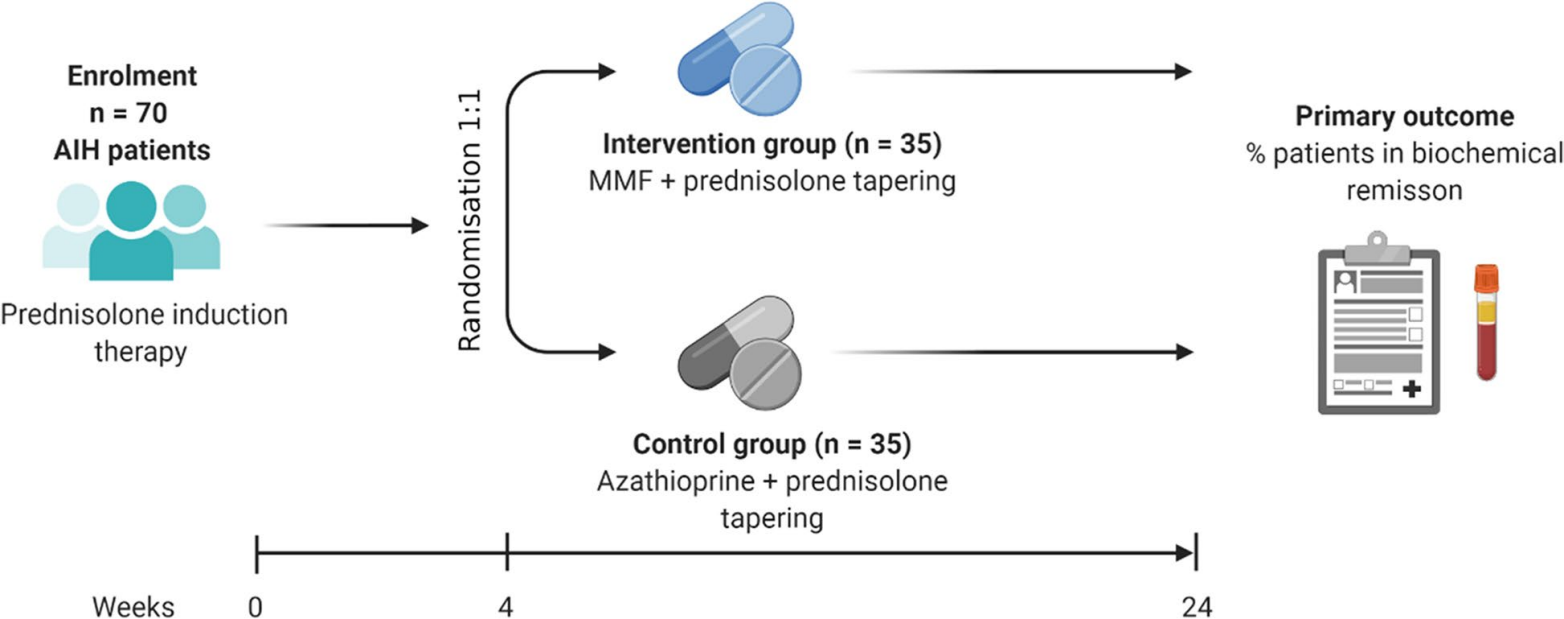
Flowchart CAMARO trial. AIH, autoimmune hepatitis; MMF, mycophenolate mofetil

CAMARO is a phase IV, randomised (1:1), open-label, parallel-group, multicentre superiority trial in 70 patients with treatment-naive AIH. All patients with established AIH according to the simplified criteria for the diagnosis of AIH [32] and who are considered for induction therapy will be eligible for the study. Eligibility will be assessed at the screening visit (SV) and patients will start with prednisolone according to the treatment schedules in Tables 2 and 3. A patient may enter the study at any time point in the first 4 weeks, as long as the prednisone schedule has been used for induction therapy. After informed consent, patients will be randomised in a 1:1 ratio to one of the two treatment arms. At 4 weeks, patients will consequently start with either azathioprine or MMF in dosages exactly according to the schedule (Tables 2 and 3). Eight follow-up visits are scheduled at weeks 4, 8, 12, 16, 20, and 24. The week 24 visit is the end of the treatment visit. After that, patients can continue azathioprine or MMF as assigned, as maintenance therapy. A trial flowchart is shown in Fig. 1.

**Table 2:** Treatment schedule for patients 40–80 kg

Week	Prednisolone (mg/day)	Azathioprine (mg/day)	MMF (mg/day)
1	40	-	-
2	30	-	-
3	25	-	-
4	20	-	-
5	15	50	1000
6	12,5	50	1000
7+8	10	100	2000
9+10	7,5	100	2000
From week 11 onwards	5	100	2000

*MMF* mycophenolate mofetil, *mg* milligrams

**Table 3:** Treatment schedule for patients >80 kg

Week	Prednisolone (mg/day)	Azathioprine (mg/day)	MMF (mg/day)
1	60	-	-
2	45	-	-
3	30	-	-
4	20	-	-
5	15	50	1000
6	12,5	50	1000
7+8	10	100	2000
9+10	7,5	100	2000
From week 11 onwards	5	100	2000

*MMF* mycophenolate mofetil, *mg* milligrams

Patients will be selected from outpatient clinics of the participating centres or referred by other centres. This is the predominant way of recruitment. Flyers with a brief introduction to the trial and researchers’ contact information will also be used for recruitment. Physicians can contact the study coordinators via telephone 24 h per day, seven days per week, to check the eligibility criteria. Potential participants will be approached by their treating physician. The study will be explained by the local Principal Investigator (PI) and their team. Potential participants received a Patients Information Form (PIF) and will have the possibility to ask questions. They have at least one week to consider the study. After one week, potential participants will be contacted, and they will get the opportunity to ask questions. The screening evaluation includes disease-related signs and corresponding laboratory tests, including a urine pregnancy test (in women of childbearing age). In some participating centres, informed consent forms will include the option to consent to collect and use biobank blood samples. The blood samples will be drawn at screening and week 24 for future research. The biobank samples will be processed locally and stored in local biobank facility. Additional tests can be performed when new research questions arise during the study. Where possible, backup samples for central endpoint analysis were drawn and stored locally.

### Participants

The objective is to include 70 patients with treatment-naive AIH. The study will be conducted by the Dutch Autoimmune Hepatitis Working Group. Individuals who meet the entry criteria and complete the baseline visit will be enrolled in one of the participating centres. In total, fourteen centres are participating in the trial, including seven Dutch university medical centres. A list of study sites can be obtained at https://clinicaltrials.gov/ct2/show/NCT02900443.

#### Inclusion criteria

The inclusion criteria are:

1. Probable or definite diagnosis of AIH according to the simplified criteria for the diagnosis of Autoimmune Hepatitis [32]:
  Definite AIH: ≥7
  Probable AIH: 6
2. First presentation of AIH requiring treatment according to the current EASL guidelines [27]
3. Age ≥18 years

All patients will undergo a liver biopsy since this is a prerequisite for the diagnosis of AIH. The liver biopsy must be scored at least compatible or typical for AIH according to the simplified criteria [32].

#### Exclusion criteria

A potential participant who meets any of the following criteria will be excluded from participation in this study:

1. Variant syndrome with primary sclerosing cholangitis (PSC) or primary biliary cholangitis (PBC) (Paris criteria [33], strong positive AMA, past liver biopsy with characteristic signs of PBC or cholangiographic findings compatible with PSC)
2. Presentation with acute liver failure, defined as presence of hepatic encephalopathy and coagulopathy (international normalised ratio (INR) > 1.5)
3. Current treatment with predniso(lo)ne and/or immunosuppressive medication for an indication other than AIH
4. Current systemic infection
5. Other clinically significant medical conditions that could interfere with the trial
6. If female of childbearing potential: known pregnancy, or unwilling to practice anticontraceptive measures
7. History of noncompliance with medical regimens, or patients who are considered to be potentially unreliable or unable to participate
8. Mental instability or incompetence, such that the validity of informed consent or compliance with the trial is uncertain

### Withdrawal from the study and replacement

Participants can leave the study at any time for any reason if they wish to do so without any consequences. The investigator can decide to withdraw a subject from the study for urgent medical reasons, such as (but not limited to) (a) occurrence of any AE or abnormality in a laboratory assessment which warrants the subject’s discontinuation from the trial, (b) patient noncompliance defined as refusal or inability to adhere to the trial schedule or procedures, (c) at the request of a regulatory authority, (d) the patient becomes pregnant, and (e) or the patients is lost to follow-up. If so, the patient will be followed and data will be recorded in an observational manner. All data generated up to the time of discontinuation from the study will be used for the intention to treat analysis and the reason(s) for discontinuation will be recorded.

Patients who participate in the study, but drop out for the study in the first 4 weeks will be replaced by new patients. If the participant is being withdrawn because of an AE, that AE must be indicated as the reason for withdrawal. Participants who withdraw from the study will be seen for a follow-up 4 weeks later. AEs and concomitant medication will be assessed and blood will be drawn.

### Treatment groups and randomization

All participants, regardless of group allocation, will be treated with prednisolone induction therapy. The prednisolone dosage will commence at 40 mg or 60 mg per day, dependent on weight, and will be tapered according to a fixed schedule (Tables 1 and 2) which is based on the EASL Clinical Practice Guidelines [27]. We aim that all patients will eventually taper down to 5 mg prednisolone daily. Participants will have reduced prednisolone to 20 mg/day at week 4. Participants will be randomised 1:1, with a variable block randomization (block size 4, 6, 8) between azathioprine or MMF, stratified per participating centre for the presence of cirrhosis (yes/no). For randomisation of a patient, physicians can contact the study coordinators via telephone 24 h per day, 7 days per week in order to check the eligibility criteria and to verify whether informed consent has been obtained. Randomisation will be done by the study coordinator using the electronic case report form by Castor Electronic Data Management (Castor EDC). The study is an open-label trial which means that both patients and all research staff (including the primary caregiver and principal investigator) will be aware of the study arm allocated and thus are not blinded. Only outcome assessors will be blinded during the analyses. Therefore, unblinding will not occur in this study. Azathioprine and MMF will be packed and labelled as study medication according to Good Manufacturing Practice (GMP) guidelines by the pharmacy of the Leiden University Medical Center.

#### Intervention group: mycophenolate mofetil (MMF)

Patients in the intervention group will receive oral MMF in a total daily dose of up to 2000 mg, split-dose, in addition to a prednisolone schedule, based on the EASL guidelines [27]. MMF will be started in a dosage of 500 mg, twice daily combined with prednisolone. If there are no problems with tolerability of MMF, patients will be titrated after 2 weeks to 1000 mg twice daily for the rest of the study. In case of MMF-related AEs (e.g. cytopenia), dose reduction can be considered by the treating physician. The dosage will be reduced with 250 mg twice daily until AEs disappear. When AEs persist after dose reduction, MMF can be discontinued. Those patients will be followed-up after discontinuation and will be treated according to standard of care by the treating physician. The teratogenic effect of MMF will be discussed in detail in women of childbearing potential and men who wish to father a child at the screening visit. All female patients of childbearing potential will need to undergo a pregnancy test at screening and will be instructed to take adequate anticontraceptive measures during the study.

#### Control group: azathioprine

The control arm will receive standard of care treatment for AIH according to the recent EASL guidelines [27]. The prednisolone dosage is based on these guidelines and is identical to the intervention group. In addition to prednisolone, standard therapy consists of azathioprine in a dosage of 50 mg daily for the first 2 weeks, and 100 mg daily for the rest of the study. The patients in this group will undergo the same study schedule as the patients in the intervention group. In case of azathioprine intolerance and/or AEs, a dosage reduction is allowed from 100 mg to 50 mg daily. If AEs related to azathioprine persist at a dosage of 50 mg, discontinuation of the drug can be considered. In that case, a patient is will be withdrawn from the study and followed up by standard of care.

#### Concomitant care

Concomitant medication may be given as medically indicated. No additional immunosuppression is allowed within the study. Participants are not allowed to participate in other (experimental) trials investigating pharmaceutical agents or strategies aimed at AIH.

#### Drug accountability

Study medication will be stored and distributed to patients according to GCP regulation and national law. Drug accountability will be performed by the (local) investigator using a drug accountability log. At the trial beginning, participants will be asked to take their medication to the clinic to control compliance. Follow-up is at least once a month to actively remind patients.

### Outcomes

#### Primary outcome

The primary outcome is the percentage of patients in biochemical remission, defined as normalisation of serum ALT and IgG levels, per treatment group. This primary endpoint will be assessed at 24 weeks of treatment.

#### Secondary outcomes

The secondary outcomes are to evaluate the efficacy of MMF versus azathioprine in combination with a 24-week prednisolone taper regimen in patients with AIH measured by the following:

- Biochemical remission at 24 weeks and at anytime
- Time to biochemical remission
- Complete biochemical response: defined as normalisation of AST, ALT, and IgG at 24 weeks
- Non-response at 4 weeks: defined as <50% decrease of serum transaminases within 4 weeks after initiation of treatment
- Insufficient response: defined as lack of complete biochemical response determined at 24 weeks
- Changes in MELD score (and its components bilirubin, INR, creatinine), and in albumin
- Changes in liver stiffness, measured by transient elastography
- N-terminal procollagen-III-peptide, Enhanced Liver Fibrosis (ELF) score
- Changes in quality of life measured with Short Form (SF)-36
- Difference in side-effects, AEs and serious AEs (SAE), based on clinical examination (e.g. hypertension), laboratory assessments (e.g. neutropenia and new-onset diabetes), and patients-reported symptoms between the MMF and azathioprine groups
- The level of ALT, AST, gamma-glutamyl transferase (GGT) in both groups
- Percentage of patients with biochemical remission
- Ratio of ALT to lowest ALT ever
- Extrahepatic AIH manifestations (e.g. arthralgia)
- Patient survival
- Fatigue index
- Pruritis visual analogue score
- Difference in cumulative corticosteroid dose between the MMF and azathioprine groups

Ancillary studies can be performed on blood samples as determined by the Dutch AIH Group steering committee.

#### Other outcomes

Other study parameters include SAEs, physical examination, and clinical laboratory tests. At every study visit, blood samples will be drawn. This will be done according to the schedule of assessments at weeks 0, 4, 8, 12, 16, 20, and 24 (Table 4). Laboratory testing will be done locally. The following tests will be standardly performed: haemoglobin (Hb), haematocrit (ht), absolute neutrophil count, white blood cell count, platelet count, prothrombin time, INR, ALT, AST, GGT, albumin, alkaline phosphatase (ALP), bilirubin (direct and indirect), creatinine (eGFR), IgG, and urinalysis: pregnancy tests in women of childbearing potential. All patients will undergo transient elastography by a Fibroscan at screening or at week 4, and at week 24 for assessment of liver fibrosis.

**Table 4:**
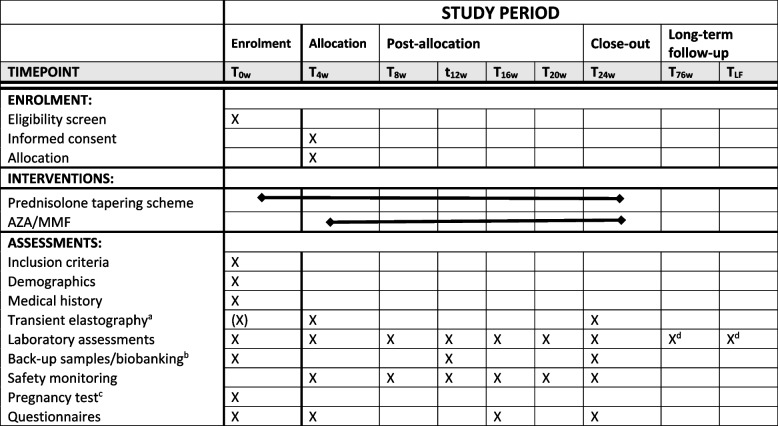
The schedule of enrolment, interventions, and assessments in the Standard Protocol Items: Recommendations for Interventional Trials (SPIRIT) figure

*AIH* autoimmune hepatitis, *AZA* azathioprine, *LF* last known follow-up, *MMF* mycophenolate mofetil

^a^Done in week 4 if not available in participating centre ^b^optional ^c^in women of childbearing potential ^d^retrospective collection of data

It is expected that patients will continue to have regular visits outside of the study context. In this natural cohort of patients, data regarding the continuation of study medication, experienced side effects and persisting remission will be collected 52 weeks after the end of the study visit (T76) and at the last known follow-up. This data will be collected retrospectively and outside of the study context.

#### Patient-reported outcomes

Questionnaires will be filled in at screening, 4, 16, and 24 weeks. Quality of life will be assessed using the SF-36, and the Work Productivity and Activity Impairment Questionnaire: General Health V2.0 questionnaires [34, 35]. AIH-related complaints will be assessed using the Liver Disease Symptom Index 2.0 questionnaire [36].

#### Sample size calculation

Since most evidence from the standard AIH treatment comes from clinical trials performed several decades ago, we have used the more recent budesonide trial from Manns et al. to determine efficacy rate of prednisolone and azathioprine in AIH [37]. In this study, 38.8% of patients reached normalisation of AST and ALT levels after 6 months of prednisolone and azathioprine treatment. When it comes to treatment with MMF, only limited data is available. The only prospective study showed that 88% of patients reached normalisation of ALT, AST, and IgG within 1-12 months, while at three months 70% reached normalisation [28]. Retrospective studies show lower response rates for MMF (Table 1). Based on available literature, we hypothesise that instead of a 70% response rate at three months, 70% at 6 months with MMF is feasible in the intervention group. A sample size of 32 patients per group will results in 1-beta of 0.80 with a two-sided alfa of 0.05 significance level to detect the difference in means of −36.2% (75.0% versus 38.8%). Taken into account a fallout rate of 10%, the total number in each group will therefore be 35 patients.

### Data collection

An electronic clinical record form (eCRF) will be used to collect the data on each participant. Data will be entered into a web-based eCRF (Castor EDC) from electronic patient files by site personnel or by the study coordinators. The study participants will be identified on the eCRF through their unique trial identifier, allocated at the time of allocation. All source data recorded in the eCRF will be signed by the investigator or his/her appropriate designee. Course documents include original documents related to the trial, to medical treatment and to the history of the participant. The requirements for source data for this trial will be outlined in the study monitoring plan. All investigators and physicians will comply with the requirements of the national law with regard to collection, storage, processing, and disclosure of the patients’ personal information. The trial evaluation team, who will analyse that data, consists of the coordinating investigators in consultation with the principal investigators. They will receive only anonymised data. Patients’ personal data is stored locally at each study site in locked cabinets or electronically on encrypted secure drives. Physicians who are involved in the trial will be asked to report all AEs to the coordinating investigator. Serious AEs are reported using the online module (https://www.toetsingonline.nl).

### Analysis

#### Statistical analysis

Descriptive analysis will be performed for all baseline and demographic data. Normally distributed variables will be noted as mean with standard deviation (S.D.), and not-normally distributed variables will be noted as median with range or inter-quartile range (IQR) if stated. Categorical data will be noted as numbers and percentages of the total.

All randomised patients are evaluated for primary and secondary endpoints at 24 weeks. The primary analysis is based on intention-to-treat (ITT) principles. ITT analyses will be used for all clinical outcome variables. Every patient who has received at least one dose of study medication will be included in the ITT analysis. A per-protocol analysis will also be performed in patients who received >80% of the study medication. When deemed necessary, missing data will be dealt with accordingly with supportive statistical analyses (e.g. imputation or appropriate models) to assess the potential effects. These methods will be addressed in the final manuscript. Treatment effects will be analysed by comparing the difference in proportion with biochemical remission at 24 weeks between both groups. Values will be compared using Student’s *t* test, Mann-Whitney *U* test, Pearson test, Wilcoxon’s rank sum test, Wilcoxon signed-rank test, chi-square test or Fischer’s exact test, as appropriate. Time to remission will be tested and compared using a Kaplan-Meier survival analysis with log-rank test. Time to remission will be censored at 24 weeks. Significance of statistical differences is attributed to p <0.05. All statistical analyses will be performed with Statistical Package for Social Sciences (SPSS), version 22.0.

### Monitoring and safety

AEs are defined as any undesirable experience occurring to a subject during the study, whether or not considered related to the investigational product. All AEs reported spontaneously by the subject or observed by the investigator will be recorded. A SAE is any untoward medical occurrence or effect that (a) results in death; (b) is life-threatening (at the time of the event); (c) requires hospitalisation or prolongation of existing inpatients’ hospitalisation; (d) results in persistent or significant disability or incapacity; (e) is a congenital anomaly or birth defect; and (f) required medical or surgical intervention to preclude of any other important medical event that did not result in any of the outcomes listed above due to medical or surgical intervention but could have based upon appropriate medical judgement. The investigator or participating site will report SAEs within 24 h to the coordinating investigator. The coordinating investigator will report the SAE to the medical ethical committee via Toetsingonline.nl within 7 or 15 days, dependently whether the SAE result in death or life-threatening event.

#### Auditing and monitoring

Clinical trial and data monitoring is performed by a set monitor assigned by the LUMC. The trial monitor will assess the progress of the trial at defined intervals, safety data, and critical efficacy variables and recommend to the sponsor whether to continue, modify, or terminate the trial. Auditing upon invitation of the hospital board can occur. The frequency is unknown.

#### Composition of the coordinating centre and coordinating

Leiden University Medical Center and Radboud University Medical Center are coordinating centres, where the principle investigators are employed. The coordinating investigators are located in both centres and have weekly digital meetings to discuss the study progress. They are responsible for the day-to-day support and are the first point of contact for gastroenterologists and hepatologists in the Netherlands. Additionally, the coordinating investigators will be responsible for the data analysis when follow-up is completed for all participants. The principal investigators are updated weekly by the coordinating investigators and are invited to meetings at least twice a year. The Dutch Autoimmune Hepatitis Working Group (DAIHWG) is updated twice yearly on the study’s progress during meetings. The DAIHWG consists of hepatologists with an interest for AIH from various (study) sites.

#### Amendments

All amendments, with the exception of protocol amendments necessary for the immediate elimination of hazards to study subjects, will be submitted to the medical research ethics committee of the Leiden University Medical Centre. Amendments impacting participants will be communicated with the participants concerned.

## Discussion

There is an urgent need for high-quality trials evaluating optimal therapy for AIH. The CAMARO trial addresses this clinical need and seeks an evidence base for alternative options for first-line treatment in patients with AIH. The CAMARO trial is an investigator-initiated multicentre, randomised trial comparing the efficacy and safety of MMF versus azathioprine, both combined with prednisolone, as first-line treatment in patients with AIH. Azathioprine is part of standard care, but the response is not universal, approximately 15% of patients experiencing AEs that necessitate cessation of azathioprine or changes in therapy [11]. Durable remission of AIH is needed to prevent further progression to end-stage liver disease and reduce liver-related morbidity and mortality. MMF is mainly used as second-line rescue therapy in AIH patients who are either intolerant or refractory to azathioprine. A select number of prospective studies evaluated MMF as first-line therapy in AIH [28–30]. Prospective randomised data on first-line therapy with MMF are lacking. Previous studies, mostly retrospective observational case series, have indicated that MMF is able to rescue azathioprine intolerant AIH patients, but not patients in whom azathioprine was unable to achieve remission.

In the CAMARO study, we deliberately chose to investigate the induction phase rather than maintenance of remission, as we know that AIH patients who have a rapid response to induction therapy have a better survival rate than patients without this rapid response [38, 39]. Whether this is due to disease-specific variables is unknown. In the literature, clinical trials that evaluate induction therapy in AIH are limited. It is worrisome that (a) there is a paucity of ongoing clinical trials in adult AIH, and (b) many trials are discontinued prematurely, and results are not reported. A double-blind, randomised, phase-IIb study investigated the role of budesonide in inducing remission in 203 patients with AIH. The primary endpoint was complete biochemical remission, defined as serum AST and ALT within the normal range, without steroid-specific side effects, at 6 months [37]. Another randomised study compared cyclosporine-A with prednisolone as an alternative treatment for the induction of remission, using the primary endpoint ‘complete remission’, defined as achieving AST and ALT in the normal range and the absence of any clinical signs of deterioration [40]. A recent ongoing phase II, randomised, double-blind, placebo-controlled trial investigates the role of JKB-122 as an adjunct therapy to prednisolone and azathioprine in the induction of remission (ClinicalTrials.gov: NCT04371718). A Danish research group started a clinical trial in 2008 investigating MMF versus azathioprine as a first-line treatment, similar to the CAMARO trial (ClinicalTrials.gov: NCT00687180). However, these results have not yet been published. The majority of discontinued studies were stopped due to slow enrolment (e.g. ClinicicalTrials.gov: NCT00608894, NCT04203875). Other ongoing initiatives in this field focus mainly on the use of MMF as second-line rescue therapy in AIH patients or variant syndromes (ClinicalTrials.gov: NCT04933292; NCT04376528; NCT02936596).

Only high-quality unbiased data coming from RCTs can inform future guidelines. Despite a clear strategy at the outset and taking into account a recruitment rate with a delayed start at the beginning of the trial, recruitment in the CAMARO trial has taken much longer than the initially predicted three years. A number of barriers impeded rapid enrolment. Successful recruitment in clinical trials is known to be one of the most challenging aspects in study completion, especially in the field of rare diseases. First, an international multicentric setup was needed to achieve recruitment targets. Due to various site-specific obstacles, administrative processes were met with delays (e.g. different electronic infrastructure, institutional policies, and contract requirements). Second, the choice of induction therapy is at the discretion of the attending physician. Only patients who were prescribed the fixed-dose prednisolone regimen could be included. This requirement resulted in lower inclusion rates. However, using a fixed-dose schedule instead of symptom-triggered treatment allows better comparison across arms. Third, a current challenge is that industry-driven trials in this field are on the horizon. These projects are better funded and allow for reimbursement of physician-patient interaction. We did not offer reimbursement for the time invested by physicians. In fact, it is remarkable that we have been able to conduct this study despite lacking the necessary funding. The CAMARO is an example of drug repurposing as a strategy to identify new uses for approved or investigational drugs outside the original label's scope. MMF and different second- and third-line therapies (e.g. tacrolimus, ciclosporin) have never been registered for specific use in AIH. Active presence, both onsite and on social media, low-threshold assistance with inclusion, use of newsletters for clinicians, and involvement of patient organisation(s) are of the utmost importance for awareness of a clinical trial. In order to spur enrolment, the option of a phone consultation with the study coordinators by phone (24/7) was given to verify eligibility criteria. This increased the rate of inclusions. Simultaneously, a close-knit international network of general and academic hospitals is fundamental for a successful RCT in rare diseases like AIH. The CAMARO trial is carried out by the Dutch Autoimmune Hepatitis Working Group and fourteen participating centres in the Netherlands and Belgium. Inclusion is reliant on this network.

Some limitations to the study must be considered. In the CAMARO trial, we excluded patients with variant syndromes, and results from our trial cannot be extrapolated to this population. Additionally, the primary outcome definition is slightly different from current guidelines. A recent study initiated by the International Autoimmune Hepatitis Group proposed five standardised endpoints in AIH management [38]. Our definition of biochemical remission almost aligns with the ‘complete biochemical response’ endpoint, defined as ‘normalisation of serum transaminase activity and IgG level below the ULN, and should be achieved no later than 6 months after initiation of treatment’. Despite different wording, the chosen primary endpoint (including the 6-month time frame) does include normalisation of IgG level, which is an advantage compared to previous studies [37, 40].

In conclusion, the results of the CAMARO trial will provide evidence on the efficacy of MMF relative to azathioprine in incident AIH cases.

## Trial status

The trial was registered on 14 September 2016 in the clinical trial register (URL: https://clinicaltrials.gov/ct2/show/NCT02900443). The first patient was randomised on 26 January 2017. The trial is ongoing and actively recruiting. To date, 66 patients of the 70 patients have been randomised. The original plan was to complete recruitment in 2018. However, the recruitment challenges among the different sites and the pandemic of COVID-19, have had a severe impact on our enrolments and will unfortunately result in a delayed end date.

## Supplementary Information

**Additional file 1.** Spirit checklist.

## Data Availability

Patients are coded by a numeric randomization code. Considering the ongoing nature of the study, collected data is not made publicly available. After publication of the final manuscripts, data may be available upon reasonable request. The dataset analysed during the current study and statistical code can be requested through the principle investigator of the sponsor site, as is the full protocol.

## References

[1] Pape S, Schramm C, Gevers TJ (2019). Clinical management of autoimmune hepatitis. United European Gastroenterol J.

[2] Liberal R, Vergani D, Mieli-Vergani G (2015). Update on autoimmune hepatitis. J Clin Transl Hepatol.

[3] Lv T, Li M, Zeng N, Zhang J, Li S, Chen S (2019). Systematic review and meta-analysis on the incidence and prevalence of autoimmune hepatitis in Asian, European, and American population. J Gastroenterol Hepatol.

[4] van Gerven NM, Verwer BJ, Witte BI, van Erpecum KJ, van Buuren HR, Maijers I (2014). Epidemiology and clinical characteristics of autoimmune hepatitis in the Netherlands. Scand J Gastroenterol.

[5] Czaja AJ (2009). Features and consequences of untreated type 1 autoimmune hepatitis. Liver Int.

[6] Cook GC, Mulligan R, Sherlock S (1971). Controlled prospective trial of corticosteroid therapy in active chronic hepatitis. Q J Med.

[7] Murray-Lyon IM, Stern RB, Williams R (1973). Controlled trial of prednisone and azathioprine in active chronic hepatitis. Lancet.

[8] Soloway RD, Summerskill WH, Baggenstoss AH, Geall MG, Gitnick GL, Elveback IR (1972). Clinical, biochemical, and histological remission of severe chronic active liver disease: a controlled study of treatments and early prognosis. Gastroenterology.

[9] Pape S, Gevers TJG, Belias M, Mustafajev IF, Vrolijk JM, van Hoek B (2019). Predniso(lo)ne dosage and chance of remission in patients with autoimmune hepatitis. Clin Gastroenterol Hepatol.

[10] Kanzler S, Lohr H, Gerken G, Galle PR, Lohse AW (2001). Long-term management and prognosis of autoimmune hepatitis (AIH): a single center experience. Z Gastroenterol.

[11] Pape S, Gevers TJG, Vrolijk JM, van Hoek B, Bouma G, van Nieuwkerk CMJ (2020). High discontinuation rate of azathioprine in autoimmune hepatitis, independent of time of treatment initiation. Liver Int.

[12] Giannakopoulos G, Verbaan H, Friis-Liby IL, Sangfelt P, Nyhlin N, Almer S (2019). Mycophenolate mofetil treatment in patients with autoimmune hepatitis failing standard therapy with prednisolone and azathioprine. Dig Liver Dis.

[13] Nicoll AJ, Roberts SK, Lim R, Mitchell J, Weltman M, George J (2019). Beneficial response to mycophenolate mofetil by patients with autoimmune hepatitis who have failed standard therapy, is predicted by older age and lower immunoglobulin G and INR levels. Aliment Pharmacol Ther.

[14] Roberts SK, Lim R, Strasser S, Nicoll A, Gazzola A, Mitchell J (2018). Efficacy and safety of mycophenolate mofetil in patients with autoimmune hepatitis and suboptimal outcomes after standard therapy. Clin Gastroenterol Hepatol.

[15] Baven-Pronk AM, Coenraad MJ, van Buuren HR, de Man RA, van Erpecum KJ, Lamers MM (2011). The role of mycophenolate mofetil in the management of autoimmune hepatitis and overlap syndromes. Aliment Pharmacol Ther.

[16] Jothimani D, Cramp ME, Cross TJ (2014). Role of mycophenolate mofetil for the treatment of autoimmune hepatitis-an observational study. J Clin Exp Hepatol.

[17] Wolf DC, Bojito L, Facciuto M, Lebovics E (2009). Mycophenolate mofetil for autoimmune hepatitis: a single practice experience. Dig Dis Sci.

[18] Hennes EM, Oo YH, Schramm C, Denzer U, Buggisch P, Wiegard C (2008). Mycophenolate mofetil as second line therapy in autoimmune hepatitis?. Am J Gastroenterol.

[19] Hlivko JT, Shiffman ML, Stravitz RT, Luketic VA, Sanyal AJ, Fuchs M (2008). A single center review of the use of mycophenolate mofetil in the treatment of autoimmune hepatitis. Clin Gastroenterol Hepatol.

[20] Inductivo-Yu I, Adams A, Gish RG, Wakil A, Bzowej NH, Frederick RT (2007). Mycophenolate mofetil in autoimmune hepatitis patients not responsive or intolerant to standard immunosuppressive therapy. Clin Gastroenterol Hepatol.

[21] Chatur N, Ramji A, Bain VG, Ma MM, Marotta PJ, Ghent CN (2005). Transplant immunosuppressive agents in non-transplant chronic autoimmune hepatitis: the Canadian association for the study of liver (CASL) experience with mycophenolate mofetil and tacrolimus. Liver Int.

[22] Richardson PD, James PD, Ryder SD (2000). Mycophenolate mofetil for maintenance of remission in autoimmune hepatitis in patients resistant to or intolerant of azathioprine. J Hepatol.

[23] Liberal R, Gaspar R, Lopes S, Macedo G (2021). Long-term outcome of patients with difficult-to-treat autoimmune hepatitis receiving mycophenolate mofetil. Clin Res Hepatol Gastroenterol.

[24] Devlin SM, Swain MG, Urbanski SJ, Burak KW (2004). Mycophenolate mofetil for the treatment of autoimmune hepatitis in patients refractory to standard therapy. Can J Gastroenterol.

[25] Czaja AJ, Carpenter HA (2005). Empiric therapy of autoimmune hepatitis with mycophenolate mofetil: comparison with conventional treatment for refractory disease. J Clin Gastroenterol.

[26] Mieli-Vergani G, Vergani D, Baumann U, Czubkowski P, Debray D, Dezsofi A (2018). Diagnosis and management of pediatric autoimmune liver disease: ESPGHAN Hepatology Committee Position Statement. J Pediatr Gastroenterol Nutr.

[27] European Association for the Study of the L (2015). EASL clinical practice guidelines: autoimmune hepatitis. J Hepatol.

[28] Zachou K, Gatselis N, Papadamou G, Rigopoulou EI, Dalekos GN (2011). Mycophenolate for the treatment of autoimmune hepatitis: prospective assessment of its efficacy and safety for induction and maintenance of remission in a large cohort of treatment-naive patients. J Hepatol.

[29] Zachou K, Gatselis NK, Arvaniti P, Gabeta S, Rigopoulou EI, Koukoulis GK (2016). A real-world study focused on the long-term efficacy of mycophenolate mofetil as first-line treatment of autoimmune hepatitis. Aliment Pharmacol Ther.

[30] Dalekos GN, Arvaniti P, Gatselis NK, Samakidou A, Gabeta S, Rigopoulou E (2021). First results from a propensity matching trial of mycophenolate mofetil vs. azathioprine in treatment-naive AIH patients. Front Immunol.

[31] Chan AW, Tetzlaff JM, Gotzsche PC, Altman DG, Mann H, Berlin JA (2013). SPIRIT 2013 explanation and elaboration: guidance for protocols of clinical trials. BMJ.

[32] Hennes EM, Zeniya M, Czaja AJ, Pares A, Dalekos GN, Krawitt EL (2008). Simplified criteria for the diagnosis of autoimmune hepatitis. Hepatology.

[33] Kuiper EM, Zondervan PE, van Buuren HR (2010). Paris criteria are effective in diagnosis of primary biliary cirrhosis and autoimmune hepatitis overlap syndrome. Clin Gastroenterol Hepatol.

[34] Ware JE, Sherbourne CD (1992). The MOS 36-item short-form health survey (SF-36). I. Conceptual framework and item selection. Med Care.

[35] Reilly MC, Zbrozek AS, Dukes EM (1993). The validity and reproducibility of a work productivity and activity impairment instrument. Pharmacoeconomics.

[36] van der Plas SM, Hansen BE, de Boer JB, Stijnen T, Passchier J, de Man RA (2004). The Liver Disease Symptom Index 2.0; validation of a disease-specific questionnaire. Qual Life Res.

[37] Manns MP, Woynarowski M, Kreisel W, Lurie Y, Rust C, Zuckerman E (2010). Budesonide induces remission more effectively than prednisone in a controlled trial of patients with autoimmune hepatitis. Gastroenterology.

[38] Pape S, Snijders R, Gevers TJG, Chazouilleres O, Dalekos GN, Hirschfield GM (2022). Systematic review of response criteria and endpoints in autoimmune hepatitis by the International Autoimmune Hepatitis Group. J Hepatol.

[39] Pape S, Gevers TJG, Vrolijk JM, van Hoek B, Bouma G, van Nieuwkerk CMJ (2020). Rapid response to treatment of autoimmune hepatitis associated with remission at 6 and 12 months. Clin Gastroenterol Hepatol.

[40] Nasseri-Moghaddam S, Nikfam S, Karimian S, Khashayar P, Malekzadeh R (2013). Cyclosporine-A versus prednisolone for induction of remission in auto-immune hepatitis: interim analysis report of a randomized controlled trial. Middle East J Dig Dis.

